# TIM29 is required for enhanced stem cell activity during regeneration in the flatworm *Macrostomum lignano*

**DOI:** 10.1038/s41598-020-80682-7

**Published:** 2021-01-13

**Authors:** Stijn Mouton, Kirill Ustyantsev, Frank Beltman, Lisa Glazenburg, Eugene Berezikov

**Affiliations:** 1 European Research Institute for the Biology of Ageing, University of Groningen, University Medical Center Groningen, Antonius Deusinglaan 1, 9713AV Groningen, The Netherlands; 2 Institute of Cytology and Genetics SB RAS, Prospekt Lavrentyeva 10, Novosibirsk, Russia 630090; 3 Present Address: PRA Health Sciences, Amerikaweg 18, 9407TK Assen, The Netherlands

**Keywords:** Regeneration, Stem cells

## Abstract

TIM29 is a mitochondrial inner membrane protein that interacts with the protein import complex TIM22. TIM29 was shown to stabilize the TIM22 complex but its biological function remains largely unknown. Until recently, it was classified as one of the Domain of Unknown Function (DUF) genes, with a conserved protein domain DUF2366 of unclear function. Since characterizing DUF genes can provide novel biological insight, we used previously established transcriptional profiles of the germline and stem cells of the flatworm *Macrostomum lignano* to probe conserved DUFs for their potential role in germline biology, stem cell function, regeneration, and development. Here, we demonstrate that DUF2366/TIM29 knockdown in *M. lignano* has very limited effect during the normal homeostatic condition but prevents worms from adapting to a highly proliferative state required for regeneration.

## Introduction

Protein domains of unknown function (DUFs) are often neglected, although they represent a treasure trove of unknown biology^1,2^. Domains represent the functional units of proteins and typically have distinct structures and functions. Despite decades of research, more than 20% of all domains in the Pfam database, the so-called DUFs, are still functionally uncharacterized^1,3,4^. Evolutionary conservation suggests that many of these DUFs are important, but studies indicated that they more likely represent biological functions specific to certain conditions, or certain groups of organisms, rather than being part of the core machinery common to all life^1,3^. This does not reduce their value, as their undiscovered functionality can represent novel biochemical pathways, alternative solutions to known reactions, or new regulatory mechanisms^2^. In the context of stem cell biology and regeneration, investigating DUFs can result in identifying novel aspects of the in vivo regulation of stem cells, which could provide unexpected breakthroughs for both fundamental and biomedical research. To study in vivo stem cell biology during development, adult tissue turnover, regeneration, and ageing, various model organisms are used. An increasingly attractive model is the free-living hermaphrodite flatworm *Macrostomum lignano*^5^. *M. lignano* is a transparent worm with a large mesodermal population of proliferating neoblasts, which represent flatworm stem cells and progenitors^6,7^. These neoblasts enable a high cellular turnover during adult homeostasis^8^ and a large regeneration capacity^9,10^. After amputation or incision, *M. lignano* can regenerate any part posterior of the pharynx and the anterior-most body part (the rostrum), although a head cannot be regenerated^10^. In 2016, we established transcriptional signatures of proliferating somatic neoblasts and germline cells by performing RNA-seq of FACS-isolated cells of worms in different conditions^11^. This dataset represents a convenient resource to identify DUFs with functions related to in vivo stem cell and germline regulation, regeneration, and development. It is expected that many of these regulators are conserved between flatworms and human, since about 47% of all *M. lignano* transcripts have human homologs, and it is even higher (85%) for neoblast-enriched transcripts^12^.

In this paper, we focus on one example: DUF2366. According to the Pfam database, this family of proteins is widely conserved from nematodes to humans. During our characterization of DUF2366 in *M. lignano*, two manuscripts were published, which identified DUF2366 (named C19orf53 in human) as a novel subunit of the human Translocase of the Inner Membrane 22 (TIM22) complex in HEK cells^13,14^. It was demonstrated that DUF2366 is required for maintaining the structural integrity and the assembly of the TIM22 complex, which mediates the import and insertion of hydrophobic proteins into the mitochondrial inner membrane^13,14^. Consequently, DUF2366 was renamed TIM29^13,14^. In addition, it was suggested that TIM29 contacts the Translocase of the Outer Membrane (TOM) complex, enabling transport of hydrophobic carrier substrates across the aqueous intermembrane space^13^. Interestingly, both papers studied the effect of TIM29 RNA interference (RNAi) on HEK cell proliferation, and reported contradicting results. While Kang et al. did not observe a significant effect of hTIM22 knockdown on cell proliferation^13^, Callegari et al.^14^ observed a significantly decreased cell proliferation. In other words, the importance of TIM29 for cell proliferation remained unclear.

Here, we identify the DUFs conserved in *M. lignano* and demonstrate the crucial role of Mlig-DUF2366/TIM29 for adapting to highly proliferative conditions during whole-body regeneration by means of RNA interference studies.

## Results

### Identification of uncharacterized proteins in *M. lignano*

**Figure 1:**
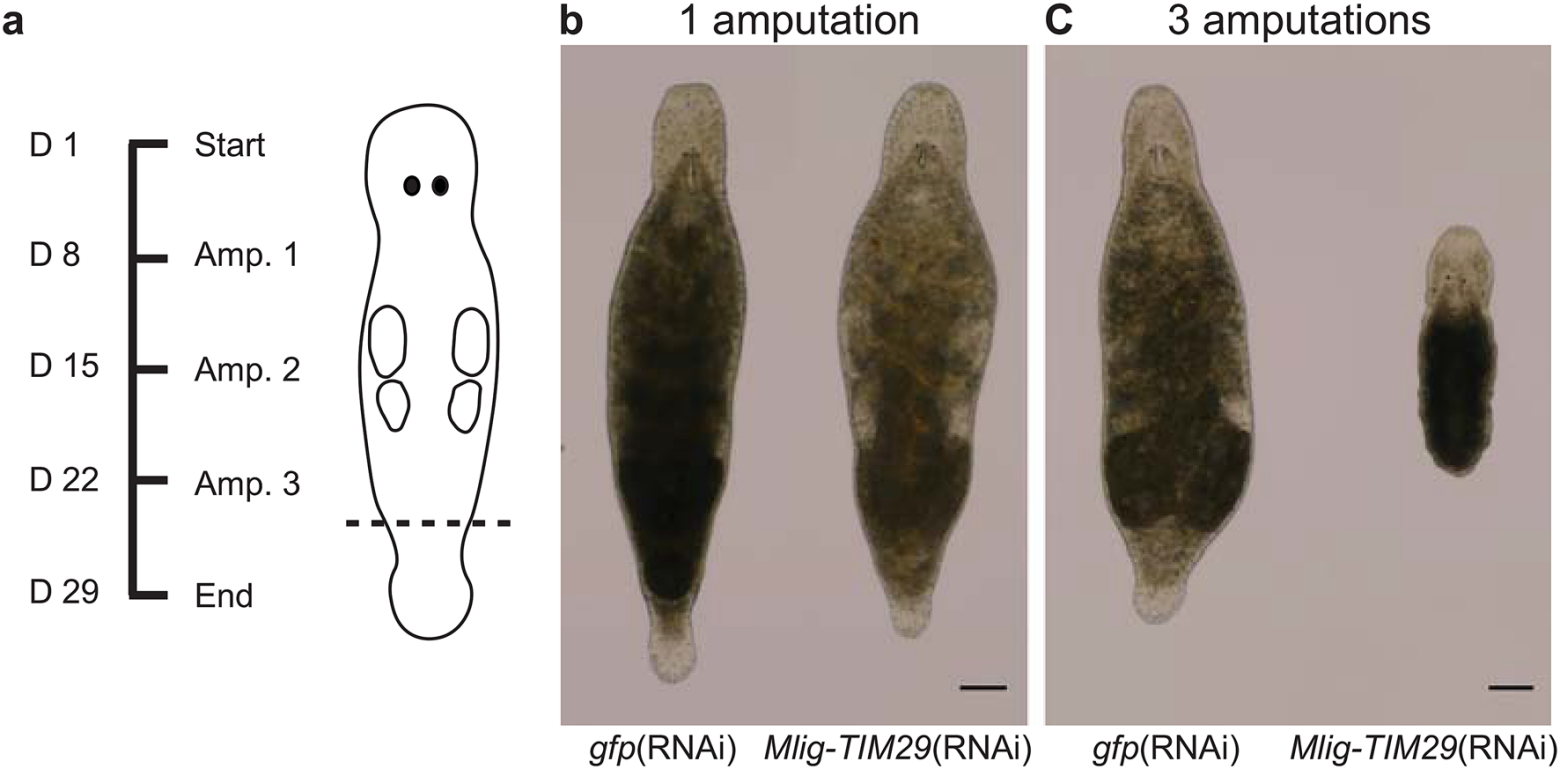
RNA interference screen. (**a**) Experimental design. D represents the time of treatment in days. Amp. describes the number of tail-amputations. The amputation plane is indicated on the worm illustration. (**b**) After a single amputation, worms are able to regenerate the tail within a week. (**c**) After the third amputation of the tail, *gfp*(RNAi) worms can regenerate, while *Mlig-TIM29*(RNAi) worms show a complete lack of regeneration. Scale bars are 100 µm.

To facilitate the discovery of novel genes involved in stem cell function, germline biology, regeneration, and development, we identified all genes in the *M. lignano* genome-guided transcriptome assembly Mlig_3_7_DV1_v3^12^ encoding uncharacterized proteins (Suppl. Table 1). Due to partial genome duplication and redundancy, very closely related genes were grouped using Corset^15^ into so-called transcript clusters for the downstream analysis^12,16^. Of the 820 identified DUF transcript clusters, 274 have identifiable homologs in human. Based on the expression level of the DUFs in different conditions and using previously established neoblast and germline transcriptional signatures^11^, categories were provided to predict their functional role in stem cells, the germline, regeneration, and development (Table 1). The value of this candidate list was tested with a pilot RNA interference (RNAi) screen of three randomly chosen genes coding uncharacterized proteins: *DUF2315* (Mlig002791.g5), *UPF0197* (Mlig006314.g7), and *DUF2366* (Mlig032364.g1). The screen focused on repeated tail regeneration of *M. lignano* (Fig. 1a), which depends on functional neoblasts. Knockdown of one of the three genes, *Mlig-DUF2366*, resulted in a reproducible phenotype. After 3 cycles of regeneration within 28 days, all (100%) *DUF2366*(RNAi) worms failed to regenerate new tissue, while all (100%) *gfp*(RNAi) worms, representing the negative control, successfully regenerated the tail (Fig. 1c). Interestingly, at least three repeated amputations of the tail-plate are necessary to induce this phenotype in 100% of the *DUF2366*(RNAi) worms. After a single tail-amputation, *DUF2366*(RNAi) worms were still able to regenerate a tail (Fig. 1b), and two tail-amputations demonstrated a variable degree of regeneration between *DUF2366*(RNAi) worms. Taken together, this suggests that without *Mlig-DUF2366* expression, worms have limited regenerative abilities. Based on these results, we decided to further characterize *DUF2366* in *M. lignano*, focusing on its requirement for stem cell function and regeneration.

**Table 1:** Categories of DUF genes in *M. lignano.*

Category	Transcript clusters^a^	DUFs
Neoblasts	13	11
Germline	36	25
Regeneration	126	78
Development	289	168
Unknown	425	198

^a^Same transcript cluster can belong to several categories.

### *M. lignano DUF2366* has an enriched expression in neoblasts and is homologous to TIM29

The *Mlig-DUF2366* gene has three nearly identical loci in the Mlig_3_7_DV1 genome assembly^12^: Mlig032364.g1, Mlig015320.g2, and Mlig018840.g2. These loci represent different gene copies emerged due to a recent whole-genome duplication and a duplication of the large chromosome of the *M. lignano* DV1 line^16,17^. All three *Mlig-DUF2366* loci have strong homology to the TIM29 protein superfamily members (Pfam, PF10171) from diverse Metazoa (Suppl. Fig. 1), and will therefore be called *Mlig-TIM29*.

The previously obtained transcriptional profiles of sorted cells^11^ demonstrated that *Mlig-TIM29* transcripts have an elevated expression in proliferating somatic neoblasts compared to differentiated cells (Suppl. Table 1). Interestingly, according to the online PlanMine resource^18^, the *Schmidtea mediterranea* homolog, dd_Smed_v6_9413_0_1 (Suppl. Fig. 1), also has a higher expression in X1 cells (cycling stem cells), compared to X2 (progenitors) and Xins cells (differentiated cells), and is included in the ‘Stem cells versus differentiated cells_low stringency’-list (Suppl. Fig. 2). This suggests that elevated expression of *TIM29* in neoblasts is conserved in multiple flatworm species. Online tools based on planarian single-cell sequencing^19–21^ further confirm that *TIM29* is predominantly expressed in clusters of neoblasts, including the cNeoblasts, and progenitors. Compared to the neoblasts/progenitors, differentiated cell-types have lower, but varying, expression levels (Suppl. Fig. 2).

The online MitoFates tool^22^ predicts that the translated protein sequence of the *Mlig-TIM29* transcripts contain a mitochondrial presequence and TOM20 recognition motifs, indicating a mitochondrial localization of the protein. MitoFates also predicts a mitochondrial presequence for dd_Smed_v6_9413_0_1 (PlanMine) and human TIM29 (GeneBank accession: NP_612367.1) translated protein sequences (data not shown).

### Experimental setup to study the role of *Mlig-TIM29* in neoblasts and regeneration

**Figure 2:**
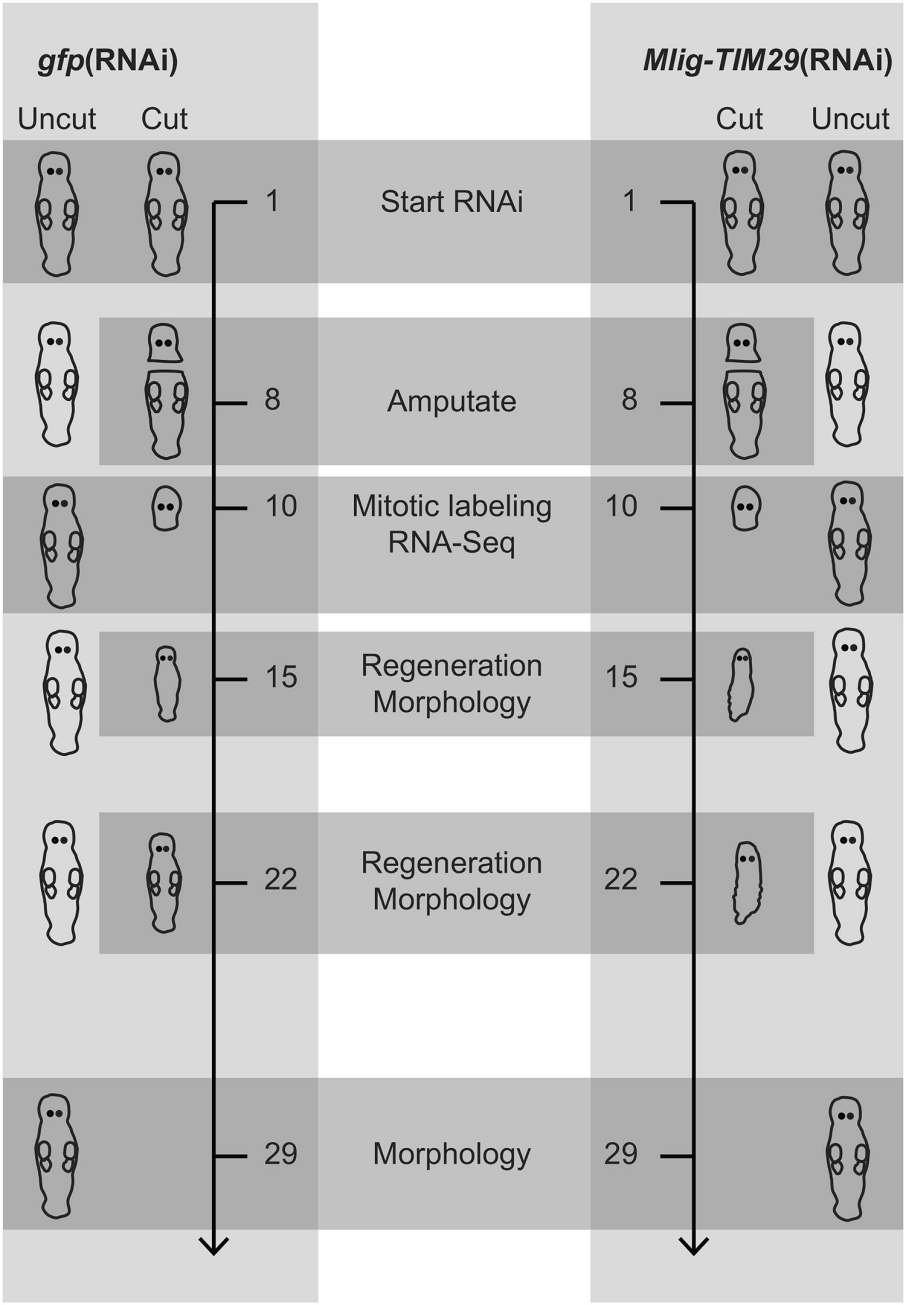
Experimental design of the *Mlig-TIM29*(RNAi) study. The horizontal grey squares indicate which worms are used at each time point, represented by the number of days.

To investigate the potential role of *Mlig-TIM29* in stem cell function and regeneration, a set of RNAi experiments was performed using *M. lignano*. In total, four specific experimental ‘classes’ were characterized: *gfp*(RNAi) uncut, *gfp*(RNAi) cut, *Mlig-TIM29*(RNAi) uncut, and *Mlig-TIM29*(RNAi) cut (Fig. 2). As GFP is not expressed in wild type worms, the *gfp*(RNAi) classes represent the negative control. The uncut conditions represent worms in which proliferation is only required for cell turnover during adult tissue homeostasis, and the production of gametes. In the cut conditions, the body was amputated by cutting worms between the pharynx and testes after 1 week of RNAi treatment, inducing regeneration of the whole body. Flatworm regeneration is a convenient readout for stem cell functionality, as it requires neoblast proliferation, migration, and differentiation.

Different measurements were performed on all four RNAi classes (Fig. 2). First, the morphology of all four classes, and the regeneration capacity of cut worms were assessed. Second, the number of mitotic cells was quantified. Third, gene expression was studied by means of RNA sequencing (Suppl. Table 2). Both mitotic labeling and RNA-seq were performed on the tenth day of RNAi, which represents the second day of regeneration in the cut worms. Fourth, to interpret the changes in gene expression, Gene Ontology (GO) Term analysis of differentially expressed genes was performed.

### *Mlig-TIM29* knockdown during homeostasis does not result in a prominent phenotype

**Figure 3:**
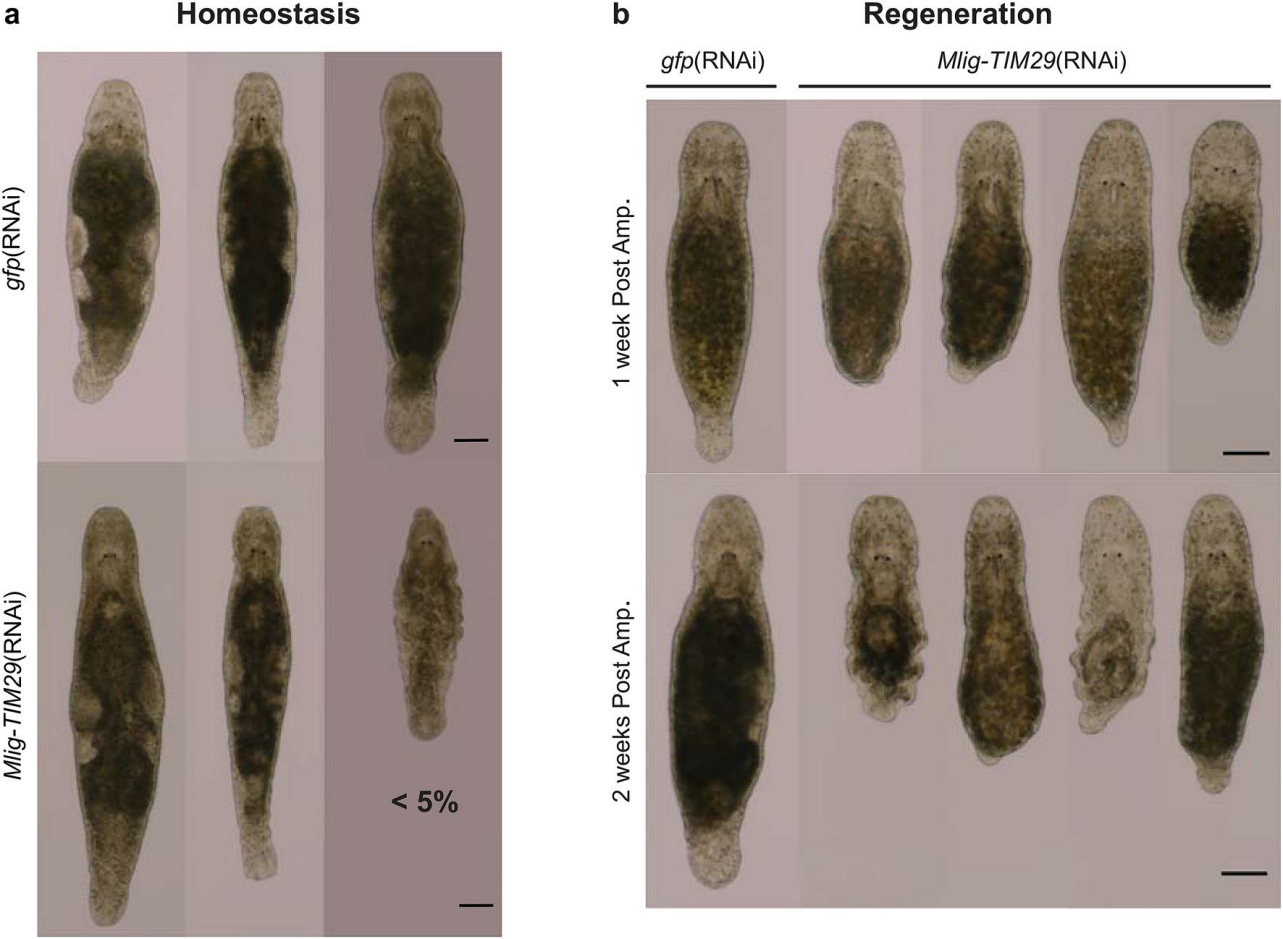
The effect of *Mlig-TIM29*(RNAi) on morphology and regeneration. (**a**) After 4 weeks of RNAi treatment, most *Mlig-TIM29*(RNAi) worms (> 95%) look similar to the *gfp*(RNAi) controls. A few individuals, however, demonstrate a decreased capacity of tissue turnover. This is illustrated by the worm on the right, which shrank, degenerated the gonads, and developed bulges in the epidermis. (**b**) During knockdown of *Mlig-TIM29* worms are not able to regenerate the body. Post Amp. represents time after amputation of the body. All scale bars are 100 µm.

**Figure 4:**
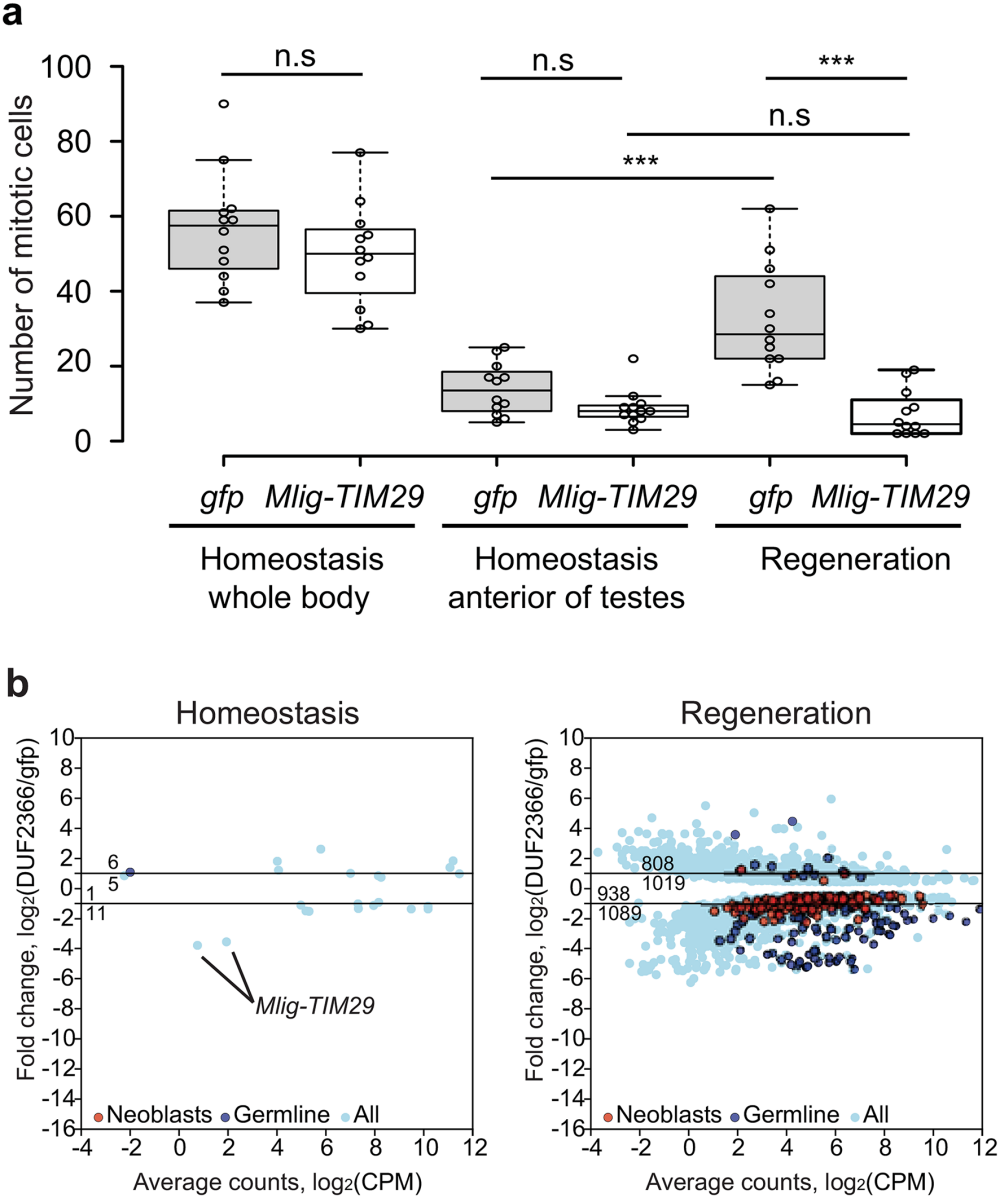
The effect of *Mlig-TIM29*(RNAi) on the number of mitotic cells and gene expression. (**a**) *Mlig-TIM29*(RNAi) causes a significant decrease in the number of mitotic cells in regenerating worms (Regeneration), but not in uncut worms (Homeostasis whole body). To allow comparison between cut and uncut worms, the number of mitotic cells was quantified in the head and pharynx region of uncut worms (Homeostasis anterior of testes) which corresponds with the fragment left after amputating the body. In control *gfp*(RNAi) worms, regeneration causes a significant increase in the number of mitotic cells. This is not observed in *Mlig-TIM29*(RNAi) worms. n.s.: *p* > 0.05, ***p < 0.001 (ANOVA and post-hoc Tukey test). (**b**) Genes differentially expressed between *gfp*(RNAi) and *Mlig-TIM29*(RNAi) worms during homeostasis (uncut worms) and regeneration (cut worms).

After 4 weeks of RNAi, the majority of uncut *Mlig-TIM29*(RNAi) worms (> 95%) still had a similar morphology as the uncut *gfp*(RNAi) worms (Fig. 3a). A limited number of individuals (< 5%), however, showed an impaired maintenance of the body by e.g. degeneration of the gonads, degeneration of the rostrum, a shrinking size, and appearance of small bulges (Fig. 3a), which often resulted in death of the individual. At the tenth day of RNAi, the number of mitotic cells, representing dividing somatic neoblasts and germline cells, was not significantly different between homeostatic control and *Mlig-TIM29*(RNAi) worms (*p* = 0.610, ANOVA and post-hoc Tukey test) (Fig. 4a, Suppl. Fig. 3). This demonstrates that *Mlig-TIM29*(RNAi) worms can maintain the proliferation rate required for cellular turnover during homeostasis. Differential gene expression analysis indicated that uncut *Mlig-TIM29*(RNAi) and uncut *gfp*(RNAi) worms only show minimal differences at the molecular level, as only 23 significantly differentially expressed genes were identified (Fig. 4b). Importantly, the two most downregulated genes, for which the expression decreased more than 10 times (log_2_ = − 3.5), correspond to the *Mlig-TIM29* transcripts. This confirms the high efficiency of the RNAi knockdown of *Mlig-TIM29*. In conclusion, efficient knockdown of *Mlig-TIM29* in uncut worms did not result in a consistent prominent phenotype within the timeframe of 4 weeks.

### *Mlig-TIM29* knockdown impairs whole-body regeneration

The regenerative ability was assessed at 1 and 2 weeks after amputation of the body, and was clearly inhibited by knockdown of *Mlig-TIM29* at both time points. One week after amputation, all *Mlig-TIM29*(RNAi) worms (100%) had a smaller tail than the negative controls, although a large variation could be observed between different individuals. In worst case, there was a complete lack of regeneration, while in best case worms regenerated a small tail plate (Fig. 3b). With advancing time, the phenotype became more apparent. Two weeks after amputation, the *gfp*(RNAi) worms were completely regenerated and resembled young adults. In contrast, all *Mlig-TIM29*(RNAi) worms (100%) were small and disproportionate. Due to impaired regenerative tissue remodeling, the location of amputation could still be observed. The morphology of the tail still varied from lacking to a small tail plate. In several cases, the appearance of bulges could be observed (Fig. 3b).

### *Mlig-TIM29* is required for adapting to regenerative conditions

The observation that knockdown of *Mlig-TIM29* results in a consistent phenotype only during triple tail-regeneration and whole-body regeneration indicates that worms cannot adapt to highly proliferative conditions during RNAi treatment. This is confirmed by differential gene expression analysis, and the quantification of mitotic neoblasts.

In *gfp*(RNAi) worms, genes with an upregulated expression due to regeneration are enriched for neoblast transcripts (1.37-fold; *p* < 10^−12^, Pearson’s Chi-squared test with Yates’ continuity correction). In contrast, in *Mlig-TIM29*(RNAi) worms, neoblast transcripts are depleted among genes with increased expression due to regeneration (0.44-fold, *p* < 10^−12^) (Suppl. Table 3). This difference indicates that *Mlig-TIM29* is required for the neoblast response during whole-body regeneration.

This observation is confirmed by quantification of mitotic neoblasts in the amputated head fragments and in the corresponding region of the body, anterior of the testes, in uncut worms. In *gfp*(RNAi) worms, regeneration causes a significant increase in the number of mitotic neoblasts, demonstrating increased proliferation during regeneration (*p* = 0.001, ANOVA and post-hoc Tukey test) (Fig. 4a). This mitotic activity is mainly located in the blastema-region (Suppl. Fig. 3). During *Mlig-TIM29* knockdown, inducing regeneration does not significantly increase the number of mitotic neoblasts (*p* = 0.999, ANOVA and post-hoc Tukey test) (Fig. 4a, Suppl. Fig. 3).

To replace missing body structures, cells in the blastema have to differentiate^23^. Both in *gfp*(RNAi) worms and *Mlig-TIM29*(RNAi) worms, genes with an upregulated expression during regeneration are enriched for GO terms related to differentiation (Suppl. Table 4). This suggests that the regenerative phenotype due to *Mlig-TIM29* knockdown is not caused by inhibited differentiation.

An important, but less understood aspect of regeneration involves anatomical remodeling to restore scale and proportion, and to allow the integration of new and old tissues^23,24^. Apoptosis has been shown to be a central mechanism for this^24^. In *gfp*(RNAi) worms, genes with an increased expression during regeneration are enriched for GO terms related to intrinsic and extrinsic apoptotic signaling pathways (Suppl. Table 4). In *Mlig-TIM29*(RNAi) worms, however, genes with a differential expression during regeneration are not enriched for GO terms related to cell death (Suppl. Table 4). These results suggest that *Mlig-TIM29* knockdown has an effect on apoptosis during regeneration, which could explain the limited remodeling. In conclusion, by impacting apoptosis and neoblast-proliferation, *Mlig-TIM29* knockdown limits the regenerative capacity.

### Knockdown of *Mlig-TIM29* has a global cellular impact in regenerating worms

**Figure 5:**
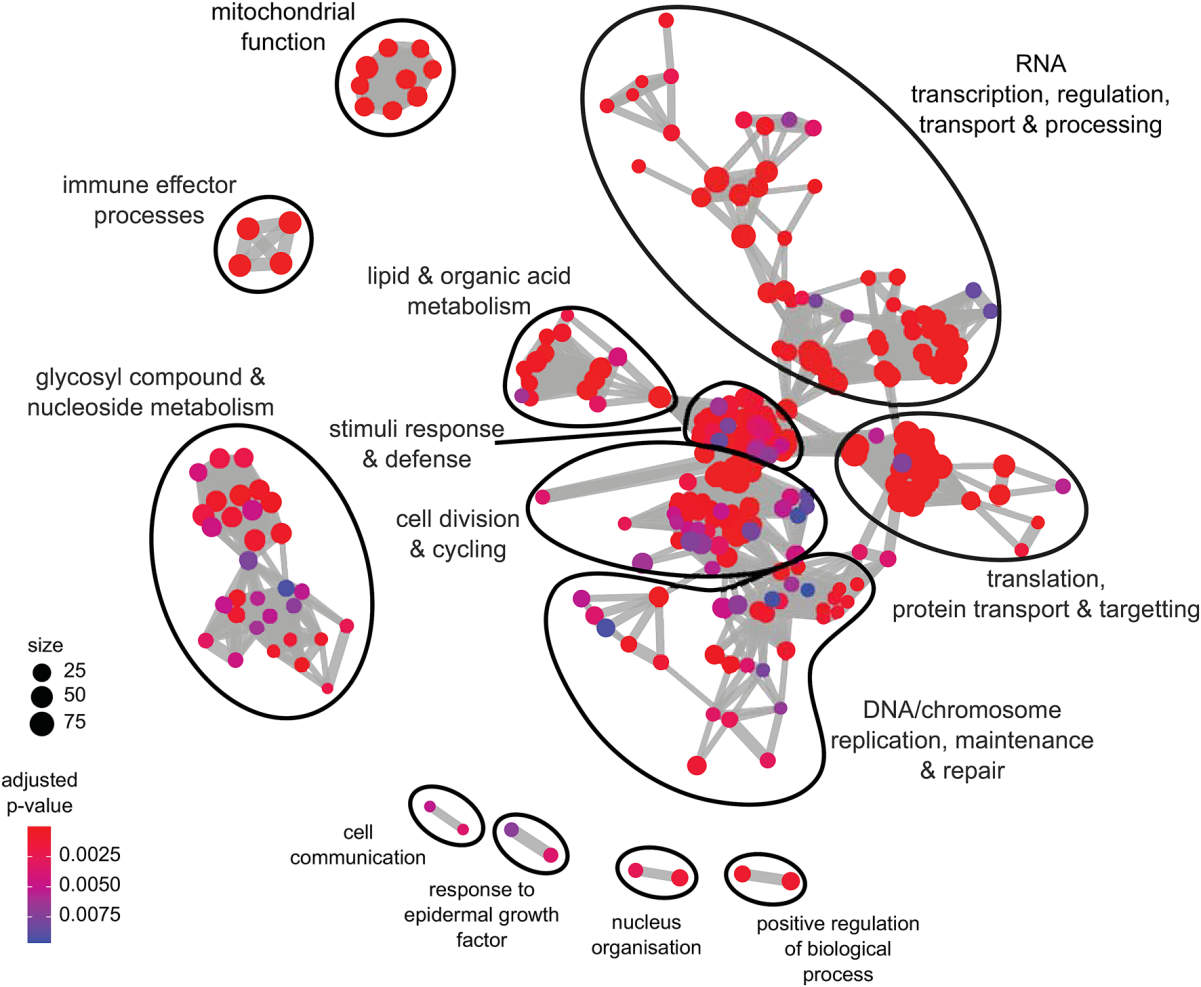
Enrichment of GO terms of biological processes categories downregulated in cut *Mlig-TIM29*(RNAi) versus cut *gfp*(RNAi) worms represented as a graph. Each GO term is shown as a circle, a node in the graph. Size of the node is proportional to the number of differentially expressed *M. lignano* human homologs assigned to the term. The nodes color corresponds to adjusted *p* values supporting its enrichment. Semantically related GO term nodes are connected with an edge. Closely related GO terms are arbitrarily outlined and given generalized names, based on the most frequent GO terms in each group.

To better understand the impaired regenerative response during *Mlig-TIM29*(RNAi), we compared gene expression between cut *Mlig-DUF2366*(RNAi) and cut *gfp*(RNAi) worms in more detail. In total, 3854 significantly differentially expressed genes were identified, of which 1827 were upregulated and 2027 were downregulated (Fig. 4b). GO term analysis shows that, during whole-body regeneration, multiple molecular biological processes are affected due to knockdown of *Mlig-TIM29.* Interestingly, however, biological clusters of significantly enriched GO terms are only found for downregulated genes. Clusters of GO terms related to cell division further confirm that *Mlig-TIM29*(RNAi) worms are not able to obtain high levels of proliferation to regenerate the body*.* In addition, large clusters of GO terms related to translation and protein transport, metabolism, transcription regulation and RNA processing, and mitochondrial function are found (Fig. 5). Thus, knockdown of *Mlig-TIM29* has a global effect, impacting multiple basic molecular processes and organelles in regenerating worms.

## Discussion

All data together demonstrates that knockdown of one gene, *Mlig-TIM29*, is enough to impair whole-body regeneration. Computational analysis indicated a mitochondrial localization of the TIM29 protein in *M. lignano*, which is consistent with the recent renaming of the human DUF2366 as TIM29 based on its characterization as an inner mitochondrial membrane protein in HEK cells^13,14^. This mitochondrial localization and function correlates with the enrichment of several GO term processes and components related to mitochondria in the differentially expressed genes between cut *Mlig-TIM29*(RNAi) and cut *gfp*(RNAi) worms (Fig. 5). The observed global changes in metabolism, translation, transcription regulation, DNA repair, stimuli response, and mitochondrial function (Fig. 5) suggest that knockdown of *Mlig-TIM29* induces a state of cellular stress which can inhibit the required molecular response for successful regeneration and growth. As a result, worms are not able to increase the proliferation rate required for whole-body regeneration, which is shown at both the cellular and molecular level (Fig. 4). In addition, GO term analysis suggests that *Mlig-TIM29* knockdown has an effect on apoptosis during regeneration, explaining the limited tissue-remodeling (Fig. 3b, Suppl. Table 4). Flatworm studies of regenerative cell death are very limited^24–26^, but it has been shown in *S. mediterranea* that regenerative apoptosis occurs predominantly in differentiated cells, and does not depend on neoblasts and their proliferation^24^. The opposite scenario, cell death triggering neoblast proliferation, has not been tested yet in flatworms^24,26^. The lack of tools makes it complicated to perform cellular studies of cell death and its crosstalk with proliferation during *Mlig-TIM29*(RNAi) and regeneration of *M. lignano* in general. Future development of transgenic tools to study diverse aspects of cells death in *M. lignano* will aid research of this important aspect of flatworm biology.

Our findings fit with the increasing recognition of mitochondrial signaling as a key component to mediate stem cell function. The emerging picture is that mitochondria continuously integrate cellular and environmental cues to influence stem cell fate and activity, which enables organisms to adapt to the environmental changes^27–29^. Many questions remain, however, as the majority of published mitochondrial research focused on post-mitotic tissues, and the role of mitochondria in the context of stem cells has been largely neglected until recent.

The development of a *Mlig-TIM29*(RNAi)-phenotype only during large scale regeneration fits with the description of DUF proteins having a function of which the importance can be limited to, or only observed during, specific conditions^1,3^. To unravel the function of DUFs, it is therefore important to study different conditions. *M. lignano* can be an appropriate model for this, as it provides an in vivo system enabling to study stem cells in their natural environment. Different conditions besides cellular turnover during adult homeostasis can be easily induced. Examples are different levels of regeneration by amputating different portions of the body, development, starvation, growth and even degrowth based on the available amount of food. Moreover, the expanding molecular toolbox and especially the recently developed methods of transgenesis will further facilitate in vivo studies to identify and characterize the function of Uncharacterized Proteins^16,30^. The here presented list of Uncharacterized Proteins presents an ideal starting point for selecting candidates. The value of this list is demonstrated as screening three candidates was sufficient for identifying a candidate which is required for adapting to highly proliferative conditions during regeneration.

## Materials and methods

### Culture of *M. lignano*

The free-living flatworm *M. lignano* is cultured in Petri dishes with nutrient-enriched artificial seawater (f/2), at a temperature of 20 °C and a 14 h/10 h light/dark cycle^31^. Worms are fed ad libitum with the diatom *Nitzschia curvilineata*^32^.

### Homology detection and sequence analysis

To find a homology to other known protein families, nucleotide sequences of the transcribed *M. lignano* DUF2366 loci as well as *S. mediterranea* dd_Smed_v6_9413_0_1 transcript sequence (PlanMine) were directly submitted to the NCBI Conserved Domain Search server^33^. Open reading frames (ORFs) analysis, multiple sequence alignments construction and visualization were done in Uinpro UGENE v34.0^34^. Amino acid sequences of TIM29 conserved domain family of other Metazoa species were obtained directly from Pfam (https://pfam.xfam.org/family/PF10171). Prediction of mitochondrial processing presequence and TOM20 recognition motifs was performed using the online MitoFates tool^22^ submitting translated ORFs sequences.

### RNA interference

The production of dsRNA was performed following a previously published protocol^11,12^, and the primers of candidate genes are presented in (Suppl. Table 5). Candidate genes were knocked down by means of RNAi with double-stranded RNA delivered by soaking as previously described^11,35^. The RNAi soaking experiments were performed in 24-well plates in which diatoms were grown. Individual wells contained 300 µl of dsRNA solution (10 ng/µl in f/2 medium) in which 15 individuals were maintained. The preliminary RNAi-screen, lasted for 4 weeks, and the tail of worms was amputated after 1, 2, and 3 weeks of RNAi treatment. For the *Mlig-TIM29*(RNAi)-screen, worms of the regenerative condition were treated for 3 weeks, and the body was amputated by cutting the worms in the region between the testes and pharynx after 1 week of RNAi treatment. Worms of the homeostasis condition were treated for 4 weeks. In all RNAi treatments, animals were weekly transferred to fresh 24-well plates to ensure sufficient amounts of food. As a negative control, *gfp* dsRNA was used. To quantify the occurrence of phenotypes, a stereomicroscope was used to observe worms, and 3 independent RNAi experiments starting with 15 worms (total n = 45) were performed for each condition: *gfp*(RNAi) uncut, *gfp*(RNAi) cut, *Mlig-TIM29*(RNAi) uncut, and *Mlig-TIM29*(RNAi) cut. To illustrate the observed changes in morphology and regeneration capacity, photos were taken using an EVOS XL Core Imaging System (ThermoFisher). For this, worms were temporary relaxed in 1:1 f/2:MgCl_2_.6H_2_O (7.14%) in a small drop in a Petri dish.

### Mitotic labeling

For both *Mlig-TIM29*(RNAi) and *gfp*(RNAi), cut and uncut worms were collected at the tenth day of RNAi treatment. In the cut condition, this time point represents 2 days after amputation. Mitotic labeling was performed as described before^6,11^. In short, worms were washed in f/2 medium, relaxed in 1:1 f/2:MgCl_2_·6H_2_O (7.14%), and fixed in 4% paraformaldehyde (PFA) for 1 h at room temperature (RT). Afterwards, they were washed with PBS-T (PBS and 0.1% Triton X-100) and blocked with BSA-T (1% bovine serum albumin in PBS-T) for 30 min at RT. The primary anti-phospho histone H3 Antibody (Millipore) was diluted 1:250 in BSA-T and applied overnight at 4 °C, followed by washing with PBS-T at RT. Worms were then incubated in secondary goat anti-rabbit IgG Antibody conjugated with FITC (Millipore) which is diluted 1:150 in BSA-T, for 1 h at RT. After being washed with PBS-T, slides were mounted using Vectashield (Vector Laboratories US, Burlingame, CA). Mitotic cells were visualized using a Leica TCS SP8 confocal microscope and were counted through the entire Z-stack of the animals using the Cell counter plugin in ImageJ. For each of the four conditions, the number of mitotic cells was quantified for a total of 12 individuals obtained from two independent labeling-experiments (n = 2 * 6). To determine if the number of cells was significantly different between conditions, an ANOVA and post-hoc Tukey test were performed in SPSS.

### Preparation and sequencing of RNA-seq libraries

Worms of the four different conditions (*Mlig-TIM29*(RNAi) uncut; *Mlig-TIM29*(RNAi) cut; *gfp*(RNAi) uncut; *gfp*(RNAi) cut) were collected at the tenth day of RNAi treatment. In the cut condition, this time point represents 2 days after amputation. For each condition, four replicates of 45 individuals each were rinsed with f/2 medium, suspended in 500 µl TRIzol reagent (Ambion) and stored at – 80 °C.

RNA was extracted from the samples with the Direct-zol RNA MiniPrep Kit (Zymo Research), following the manufacturer’s protocol. RNA-Seq libraries were made using the CEL-Seq2 protocol^36,37^, and as a first step a mix of RNA, primer, spike-in, and dNTPs was made. While this method was originally designed for single cells, it also works well with larger amounts of RNA. Sequencing was performed using the T-fill protocol^38^ on an Illumina HiSeq 2500 machine.

### Differential expression analysis of RNA-Seq data

Illumina reads were mapped to the *M. lignano* genome assembly Mlig_3_7^16^ using STAR software v. 2.6.0c^39^ and transcriptome annotation version Mlig_RNA_3_7_v3^12^. The transcriptome quantification option of STAR was used to derive initial transcript counts, which were consolidated into transcript cluster counts using Corset^15^. Differential gene expression analysis was performed using generalized linear models implemented in edgeR software package^40^. Only transcript clusters that had at least 1 count per million in at least 3 samples were included in the analysis. FDR cutoff of 0.05 was used to establish statistically significant differentially expressed genes.

### GO Term analysis

For genes differentially expressed between various experimental conditions human homolog gene annotations were extracted from the previously annotated trascriptome Mlig_RNA_3_7_v3^12^. The resulted list of *M. lignano* human homologs was then processed and analyzed using a custom script written in R programming language. Libraries “org.Hs.eg.db”^41^ and “clusterProfiler”^42^ were used for the GO term analysis, applying the function *enrichGO* with the following parameters: [List of Human ENTREZ gene IDs], OrgDb = org.Hs.eg.db, ont = “BP”, pvalueCutoff = 0.01, qvalueCutoff = 0.01, pAdjustMethod = “BH”. The results of the analysis were visualized as a graph using *emapplot* function, showing all categories and coloring nodes by their adjusted p-values. The graph was exported to PDF and manually processed in Inkscape v.0.92 vector graphics software (https://inkscape.org/) to apply additional annotations.

### Data accessibility

RNA-seq data have been deposited at DDBJ/EMBL/GenBank under the BioProject accession number PRJNA606131.

## Supplementary Information

Supplementary Figures.

Supplementary Table 1.

Supplementary Table 2.

Supplementary Tables 3 and 5.

Supplementary Table 4.
