# Supplementary material for: TIM29 is required for enhanced stem cell activity during regeneration in the flatworm Macrostomum lignano
Source: Sci Rep. 2021 Jan 13;11:1166. doi: 10.1038/s41598-020-80682-7 (PMC7806878; doi:10.1038/s41598-020-80682-7)
Supplement: Supplementary file 1 — Supplementary Figures. [file 41598_2020_80682_MOESM1_ESM.pdf]

TIM29 is required for enhanced stem cell activity during regeneration in the flatworm *Macrostomum lignano*

Stijn Mouton, Kirill Ustyantsev, Frank Beltman, Lisa Glazenburg, Eugene Berezikov

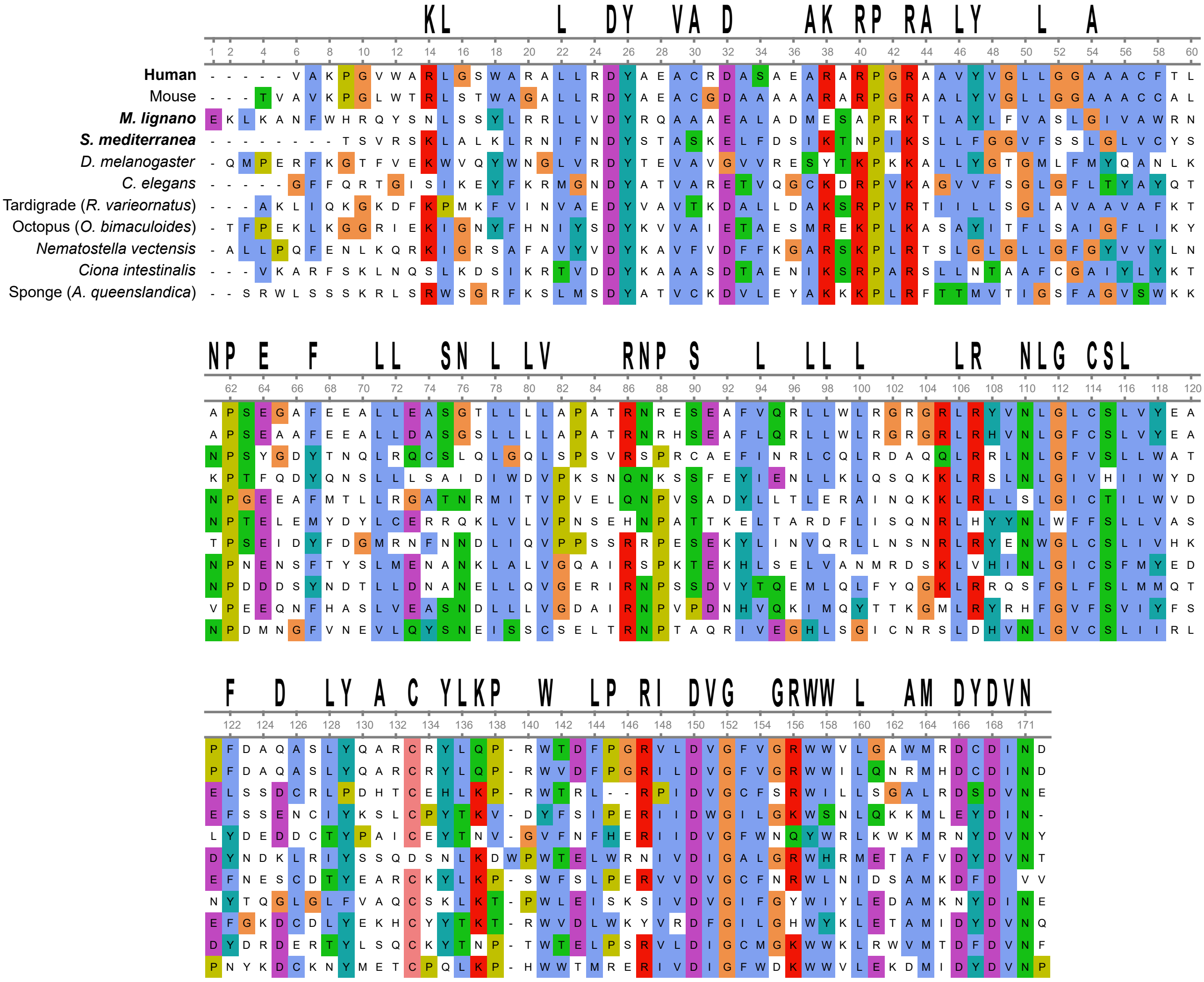

Supplementary Figure 1. Multiple amino acid sequence alignment of TIM29 family protein domains (Pfam, PF10171) from various Metazoa species. Conserved (> 50%) amino acids are designated at the top of the alignment. *M. lignano* transcript number - Mlig018840.g2. *S. mediterranea* PlanMine transcript number - dd_Smed_v6_9413_0_1. For other species, Pfam accession numbers are as follows: human - Q9BSF4, mouse - Q8BGX2, *D. melanogaster* - Q9W4R8, *C. elegans* - Q8WQD7, tardigrade - A0A1D1VF99, octopus - A0A0L8FIM2, *N. vectensis* - A7SPD9, *C. intestinalis* - H2XT55, sponge - A0A1X7SN56.

Planmine: http://planmine.mpi-cbg.de/planmine/report.do?id=9135634&trail=|9135634

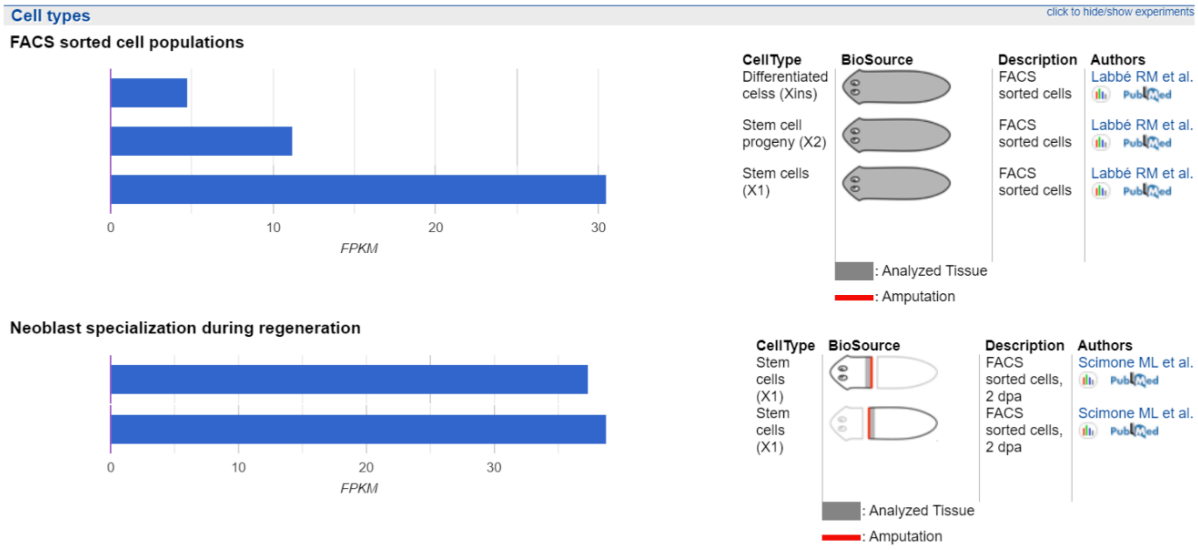

Planosphere:

https://planosphere.stowers.org/search/page/transcript

Reference Sequence Information			Observation Curated from Publication			
ID	Description	Gene Models	Seq ID	Expressed In	Experimental Details	PMID
SMED30007386	Translocase of inner mitochondrial membrane 29	SMESG000024849.1<br>SMESG000024848.1	SmedASXL_012443	X1 cell		26114597

https://planosphere.stowers.org/feature/Schmitea/mediterranea-sexual/transcript/SMED30007386

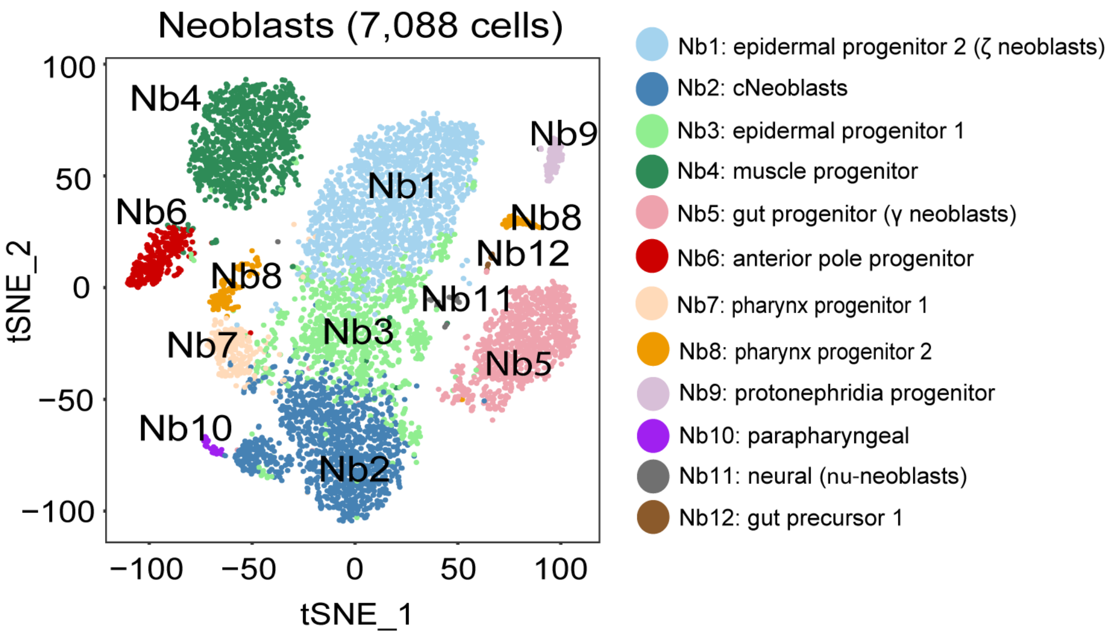

Suppl. Fig. 2. continued on the next page.

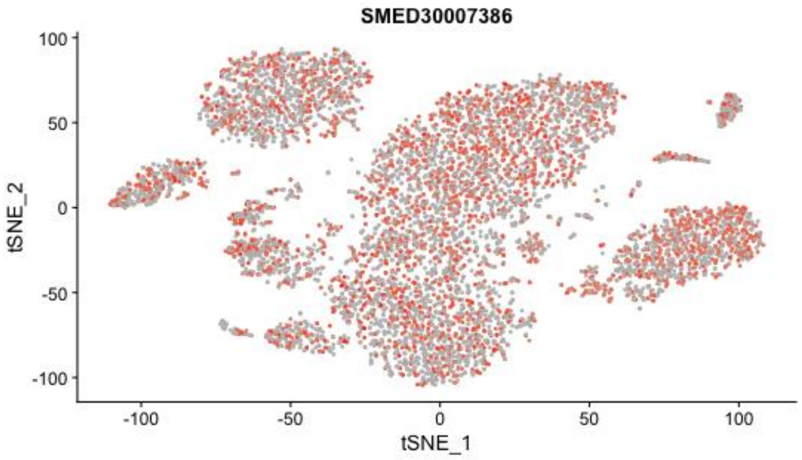

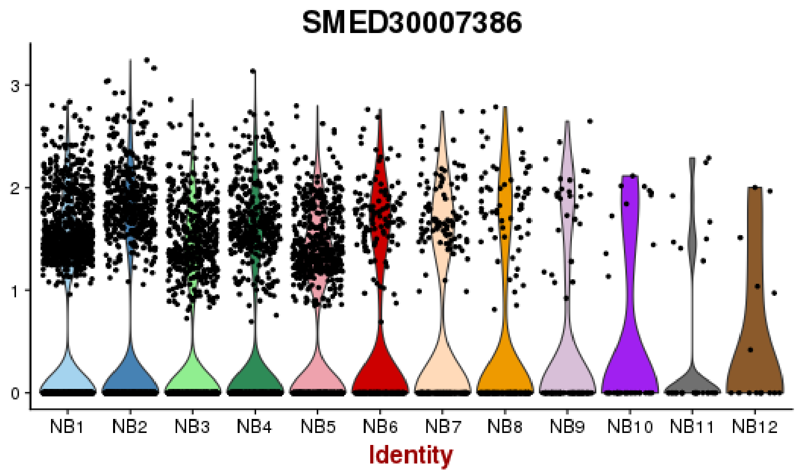

Planaria SC atlas: https://shiny.mdc-berlin.de/psca/

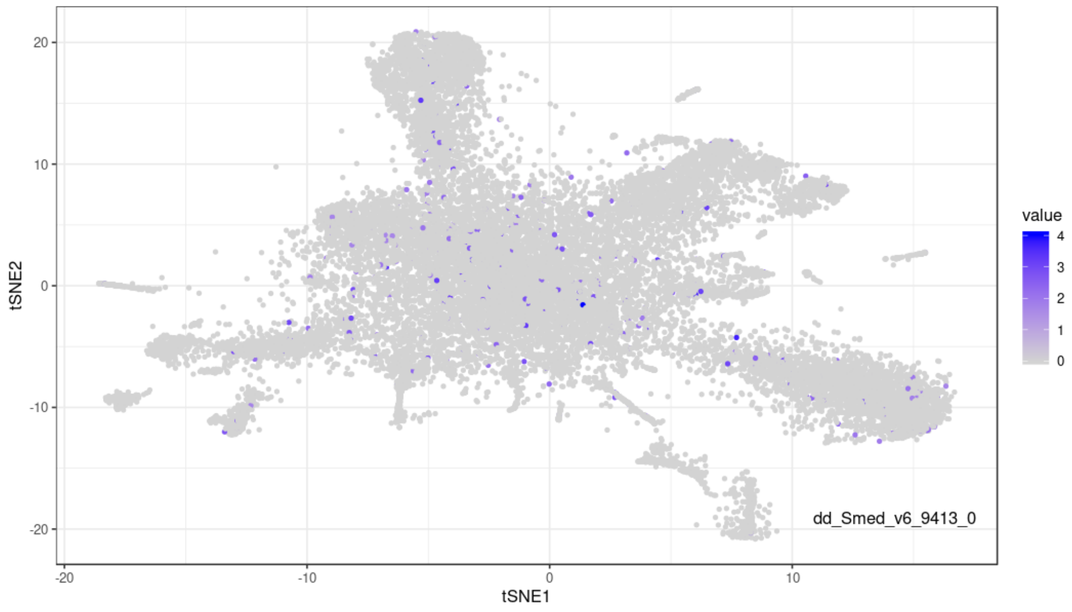

Suppl. Fig. 2. continued on the next page.

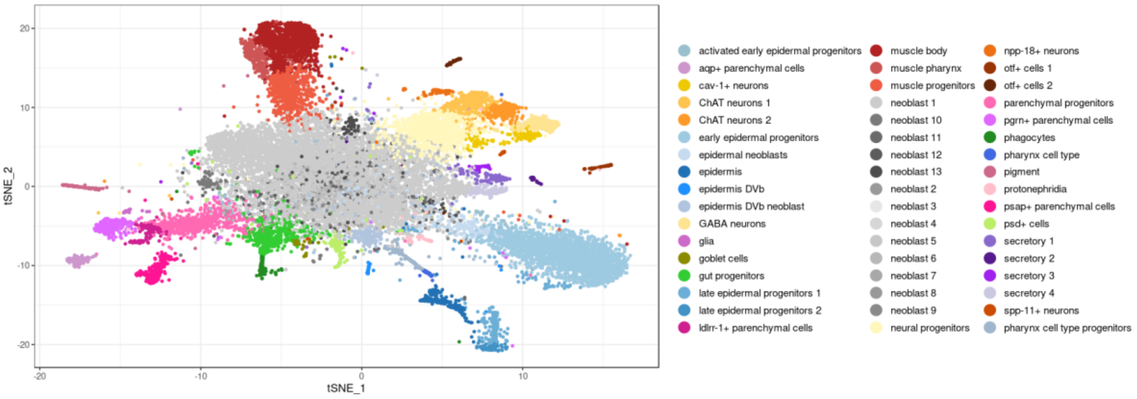

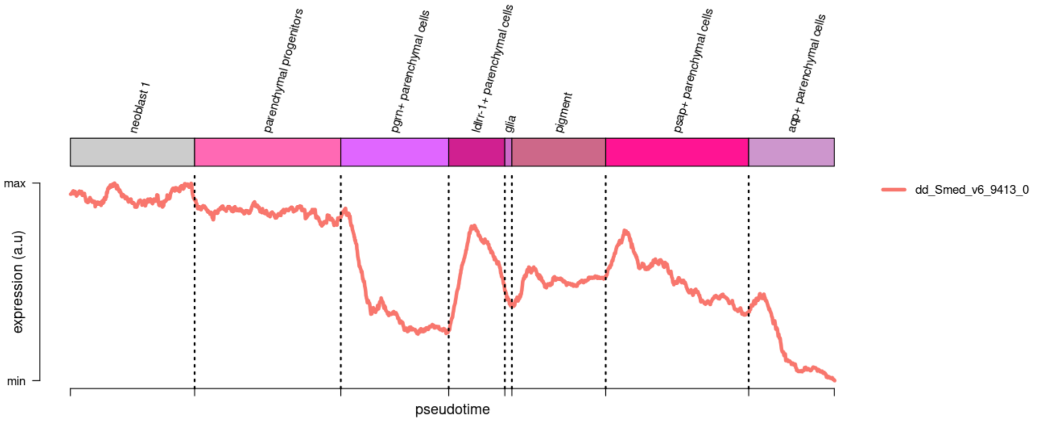

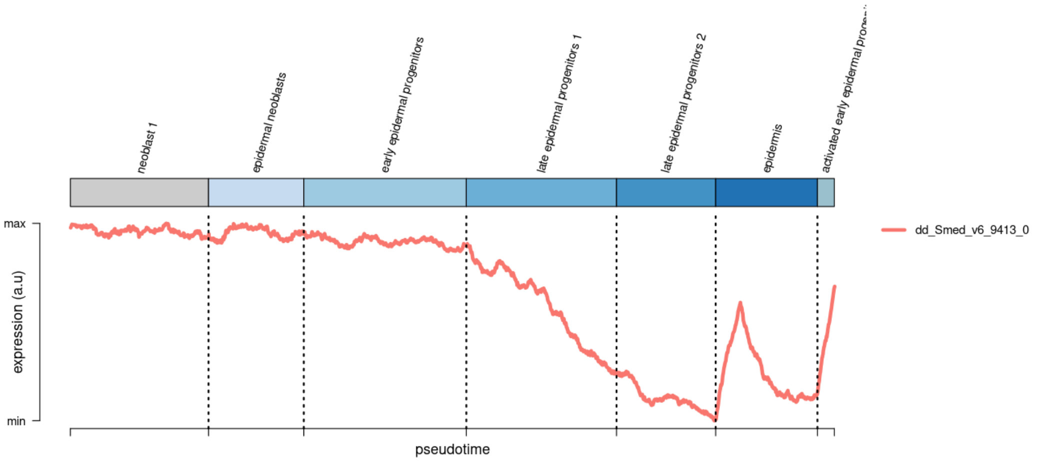

Suppl. Fig. 2. continued on the next page.

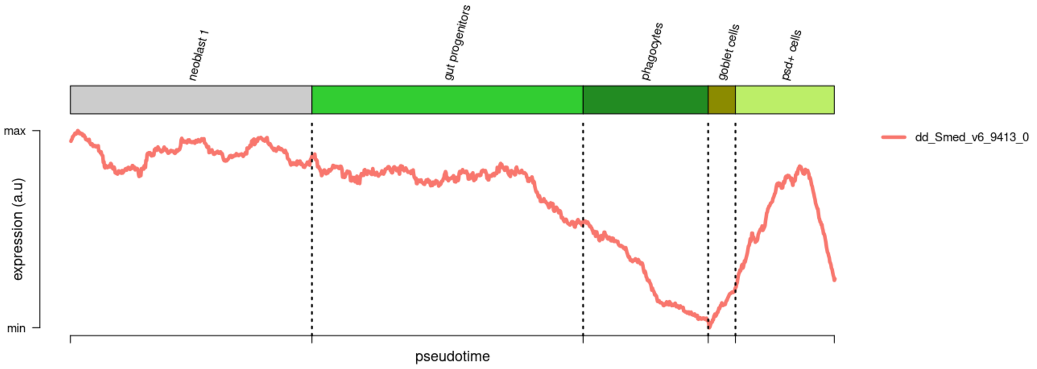

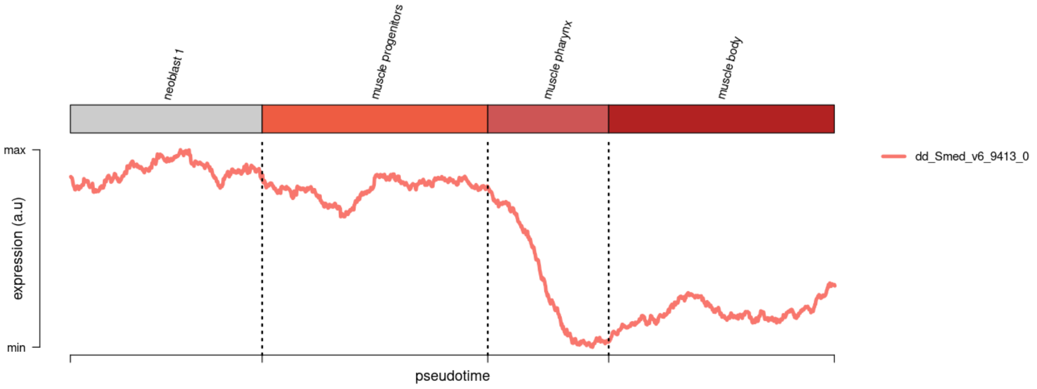

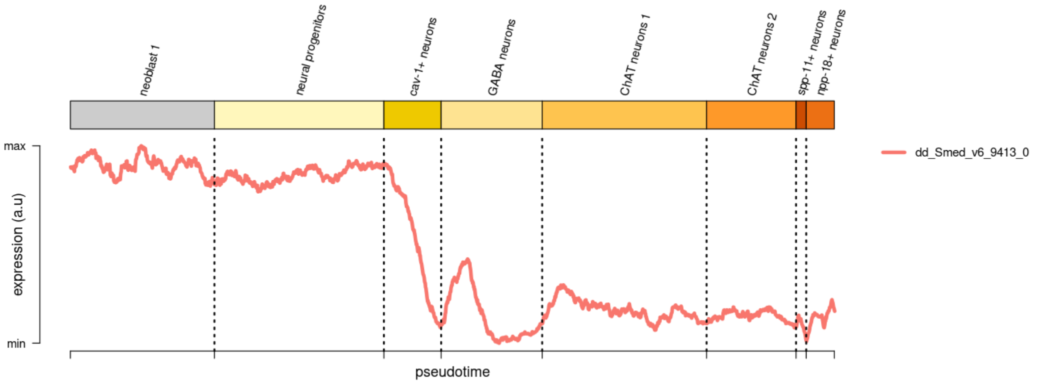

Suppl. Fig. 2. continued on the next page.

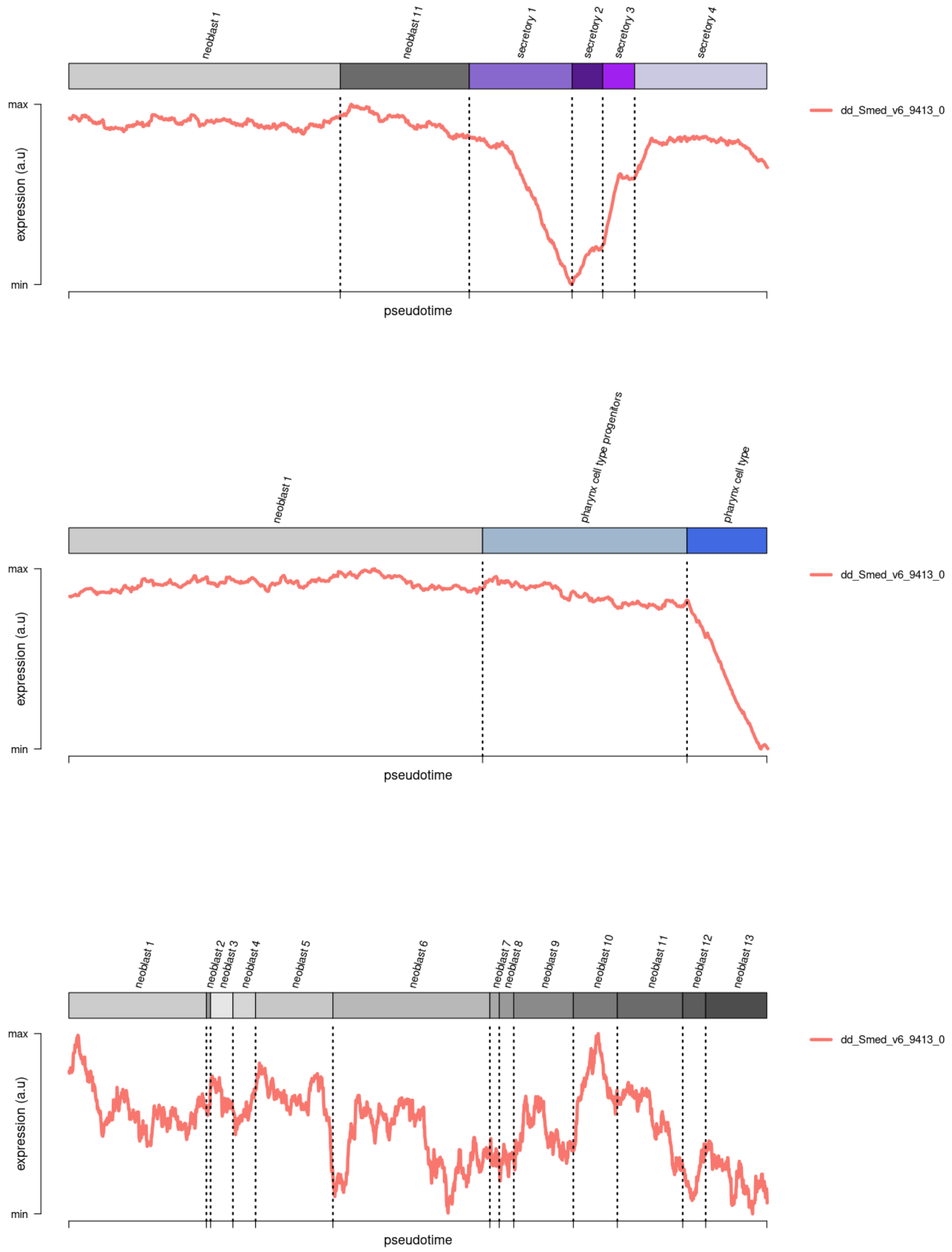

Supplementary Figure 2. TIM29 expression is elevated in, but not specific to, neoblasts. Planmine demonstrates that TIM29 has an elevated expression in proliferating X1 cells compared to X2 and differentiated cells. Planosphere indicates that TIM29 is mainly expressed in X1 cells. Expression can be observed in different neoblast-types, including the cNeoblasts and different progenitors. The Planaria SC atlas shows that TIM29 expression is elevated in neoblasts and progenitors, but not absent in differentiated cells. While most differentiated cell types have low TIM29 expression, there are some exceptions, e.g. psd+ and secretory 4 cells, with higher expression.

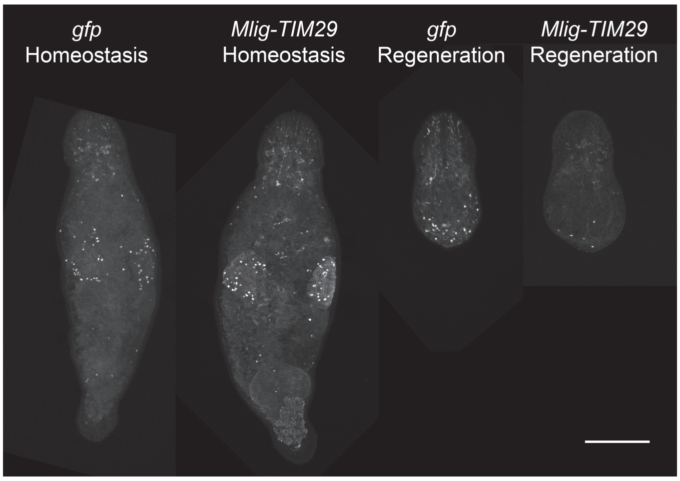

Supplementary Figure 3. Representative images of the mitotic labelling. For each RNAi-condition, a confocal z-projection of one representative worm is shown. Mitotic cells are bright and have a round/oval shape. Granular structures represent aspecific fluorescence. The scale bar is 200 µm.
