# Supplementary material for: TIM29 is required for enhanced stem cell activity during regeneration in the flatworm Macrostomum lignano
Source: Sci Rep. 2021 Jan 13;11:1166. doi: 10.1038/s41598-020-80682-7 (PMC7806878; doi:10.1038/s41598-020-80682-7)
Supplement: Supplementary file 4 — Supplementary Tables 3 and 5. [file 41598_2020_80682_MOESM4_ESM.pdf]

TIM29 is required for enhanced stem cell activity during regeneration in the flatworm *Macrostomum lignano*

Stijn Mouton, Kirill Ustyantsev, Frank Beltman, Lisa Glazenburg, Eugene Berezikov

Supplementary Table 3. Enrichment of neoblast and germline transcripts in differentially expressed genes in various comparisons.

Dataset	Expresssion Pattern	Germline	Neoblasts
TIM29 Cut compared to TIM29 Uncut	Up	0.02	0.44
TIM29 Cut compared to TIM29 Uncut	Down	5.01	1.18
GFP Cut compared to GFP Uncut	Up	0.02	1.37
GFP Cut compared to GFP Uncut	Down	5.58	0.40
TIM29 Cut compared to GFP Cut	Up	0.09	0.25
TIM29 Cut compared to GFP Cut	Down	0.68	4.77
TIM29 Uncut compared to GFP Uncut	Up	ns	ns
TIM29 Uncut compared to GFP Uncut	Down	ns	ns

Supplementary Table 5. Primers for the production of dsRNA

Gene name	Transcript ID	Forward primer	Reverse primer	Product size, nt
DUF2315	*Mlig002791.g5*	GCTTGTTATCCTCTTCATCGTC	AAGCTTATGGTTAAACCTGGTG	608
UPF0197	*Mlig006314.g7*	CACTTCTTGACCGAATGTTAAA	TTTCGGGACTAATTCAAAGTCT	658
DUF2366	*Mlig032364.g1*	AAAGTTGAAAGCAAATTTCTGG	GCCAATCGATATAAAATTGGAA	702
